# Impact of the COVID-19 pandemic on the mental health of transgender persons in India: Findings from an exploratory qualitative study

**DOI:** 10.3389/fgwh.2023.1126946

**Published:** 2023-03-15

**Authors:** Harikeerthan Raghuram, Sana Parakh, Supraja Chidambaranathan, Deepak Tugnawat, Venu Pillai, Sanjana Singh, Satendra Singh, Aqsa Shaikh, Anant Bhan

**Affiliations:** ^1^ Bhopal Hub, Sangath, Bhopal, India; ^2^ Department of Physiology, University College of Medical Sciences, University of Delhi, New Delhi, India; ^3^ Department of Community Medicine, Hamdard Institute of Medical Sciences and Research, Jamia Hamdard, New Delhi, India

**Keywords:** COVID-19, mental health, transgender, India, minority stress

## Abstract

**Introduction:**

Transgender persons in India, who are one of the most vulnerable groups, were particularly impacted by the COVID-19 pandemic. Increased risk of COVID-19, challenges with continuing livelihood, uncertainty, and anxiety around the pandemic over pre-existing social discrimination and exclusion pose risk of a mental health impact as well. To investigate this further, this component of a larger study on experiences in healthcare of transgender persons in India during COVID-19 and looks into the question “How did the COVID-19 pandemic impact the mental health of transgender persons in India”.

**Methods:**

22 In-depth interviews (IDI) and 6 focus group discussions (FGD) were conducted virtually and in-person with persons self-identifying as transgender or belonging to ethnocultural transgender communities from different parts of India. Community based participatory research approach was used by ensuring representation from the community in the research team and through a series of consultative workshops. Purposive sampling with snowballing was used. The IDIs and FGDs were recorded, transcribed verbatim and analyzed using an inductive thematic analysis.

**Results:**

Mental health of transgender persons were affected in the following ways. Firstly, COVID-19, its associated fear and suffering combined with pre-existing inaccessibility of healthcare and reduced access to mental health care affected their mental health. Secondly, unique social support needs of transgender persons were disrupted by pandemic linked restrictions. Thirdly, pre-existing vulnerabilities such as precarious employment and underlying stigma were exacerbated. Finally, gender dysphoria was a key mediating factor in the impact of COVID-19 on mental health with a negative and positive impact.

**Conclusions:**

The study reiterates the need to make systemic changes to make mental healthcare and general healthcare services trans-inclusive while also recognizing the essential nature of gender affirmative services and the need to continue them even during emergencies and disaster situations. While this brings out how public health emergencies can exacerbate vulnerabilities, it also shows how the lived mental health experience of transgender person is intricately linked to the way work, travel and housing is structured in our society and therefore points to the structural nature of the linkage between mental health and gender.

## 1. Introduction and background

Transgender persons are those persons whose experience of their gender (gender identity) is incongruent with the sex they were assigned to at birth (birth-assigned sex) (1, 2). The Census survey of 2011, which for the first time estimated the number of transgender persons in India, reports a total of 487,803 persons who identify as transgender (3). This may be an underreport as many transgender persons might have not shared their transgender identity or might have been counted in their transitioned identity as male or female. India, along with other countries in South Asia, has a unique history of people of transgender identity living together in ethno-cultural kinship communities known in various names specific to different regions. This includes the *Hijra* and *Kinnar* communities.

In India, transgender persons are among the most vulnerable population groups (4, 5). This is because of widespread marginalisation of the community which has resulted in lower levels of education, income, employment, and health status due to experiences of stigma, discrimination and violence (6). Many transgender persons in urban ethno-cultural communities are involved in begging, sex work and traditional blessing (*badhaai*) for their livelihood (6).

Transgender persons face higher rates of mental health problems such as substance use and mental health disorders including mood disorders, eating disorders, suicide ideation and behaviours (7–18). Controlled studies from the US show that transgender persons have a two-to-three-fold higher risk of depression, anxiety and suicidal ideation and attempts as compared to cisgender persons (19–21). This higher rate of mental health problems in transgender persons needs to be viewed in the backdrop of unique social determinants of mental health among transgender persons which is best conceptualized by the Minority Stress Model (22). The model posits that people belonging to minority communities face negative stressors to mental health because of stigma, prejudice and discrimination. Studies that utilize this model find that stigma, discrimination, abuse, social isolation affect mental health of transgender persons negatively and social support, peer support, parental support and gender affirming care (GAC) are beneficial (21, 23, 24).

However, despite this higher burden of mental health problems in transgender persons, they face challenges in accessing mental healthcare and healthcare in general. Challenges before, or in, reaching the health facility include low health literacy, social exclusion, financial constraints and lower education (4, 25, 26). Challenges within the health facility include discrimination, misgendering, fear of being pathologized or stereotyped, insensitivity, lack of privacy and confidentiality, trans-negative mental health professionals, and verbal and physical abuse (4, 27–31). Experiences with trans-negative health providers, in particular, have a negative impact on the mental health of transgender persons (32).

### 1.1. The COVID-19 pandemic in India

The COVID-19 pandemic started in India in early 2020 and in response the government initiated a nationwide lockdown overnight in March 2020 (33). Transgender persons have been particularly affected during the COVID-19 pandemic and its associated government responses in India, such as lockdowns, isolation and mandatory quarantine as they face an increased risk of COVID-19 due to poor housing, overcrowding, lower levels of immunity due to Hormone Replacement Therapy (HRT), Non-Communicable Diseases (NCDs) and substance use, poorer access to vaccination services and increased risk of HIV (34–47). In addition to this, the strict lockdown measures imposed in India had a particular risk to transgender persons as a large number of transgender persons are engaged in informal employment including begging, sex work and traditional blessing, all of which were difficult to pursue during the lockdown (ibid).

The pandemic has impacted mental health across low- and middle-income countries (48). In terms of its impact on the transgender community, studies from the US show that it has adversely impacted the mental health of transgender persons more than that of cisgender persons and transgender persons had a relatively higher unmet need of mental health care during the pandemic (49). The increased risk of COVID-19, challenges with livelihood combined with uncertainty and anxiety around the pandemic over existing social discrimination and exclusion of transgender persons pose increased risk of mental distress and mental health problems (45, 46). However, there is scarce data on the impact of the pandemic on the mental health of transgender persons in the Indian setting, especially covering diverse populations within the transgender community.

The TransCare COVID-19 (TCC-19) project fills this gap as it was a qualitative study focused on India and inclusive of diverse identities including transfeminine, transmasculine, gender non-binary, gender fluid, gender queer as well as people belonging to the traditional ethnocultural communities such as the *hijra* and *kinnar* communities. Conceptualized to better understand community experiences during the pandemic, TCC-19 aimed to look at what are the experiences of transgender persons in accessing and navigating healthcare in India in general and, specifically, during the COVID-19 pandemic. In this paper, we draw from data in this study to specifically look at how the COVID-19 pandemic impacted the mental health of transgender persons in India.

## 2. Methods

This paper presents results from one of the key research questions of the TCC-19 study conducted by the authors. For all the research questions, a same set of methods were used guided by a community based participatory research (CBPR) approach. The main question was about the experiences transgender persons have in accessing and navigating healthcare, especially during the COVID-19 pandemic.

In-depth interviews (IDIs) and focus group discussions (FGDs) were conducted over a period of 6 months (May 2021 to October 2021) virtually (mainly using Zoom) from across India and in-person in Bhopal, Indore and New Delhi. The respondents in our study were transgender persons who were residing in India at the time of the study. The theoretical approach of the study was based on the intersectionality approach and the theory of intersectionality of identities (50, 51) which guided the sampling, data collection and data analysis. In addition, the analysis for this paper was informed by the Minority Stress Model (22). Ethics approval for the study was obtained from the institutional review board of Sangath (AB_2021_71).

### 2.1. Use of participatory approach

The study used principles of community-based participatory research (CBPR). CBPR is an approach to co-design and co-conduct research in the context of power differences between the researcher and the “researched” that are inherent in research with marginalized populations (52, 53). CBPR was incorporated throughout the research process through several methods: participatory community workshops, an advisory board of members from the community and guidance from co-investigator AS and collaborator Sanjana Singh, who both identify as transwomen. A series of four participatory community workshops were conducted over a period of one year with the transgender community with an aim to take community inputs at all stages of the research process. Participants for the participatory component were reached out through the networks of co-authors HR, AS, Sanjana Singh and VP which made rapport and trust building easier. The first two workshops focused on co-designing the study. The third one focused on co-analysis of preliminary findings and the last one which was conducted in person focused on dissemination of study findings to plan subsequent research and advocacy. Workshops were conducted in-person or virtually over Zoom and lasted around 90–120 min. Documents were shared beforehand in Hindi and English for participants to review and then discussed during the workshop. In addition, we conducted in-person and virtual workshops to build capacity in the community in psychological support and in trans-sensitive research, respectively. The study was also guided by a consultation with an advisory board comprising of diverse persons from the transgender community. Further, where feasible we had members from the community to co-conduct the research data collection including AS and Sanjana Singh.

### 2.2. Data collection

For the study, transgender persons were defined as persons whose experience of their gender (gender identity) is incongruent with the sex they were assigned to at birth (birth-assigned sex) (1, 2). In this study our basis of identification was self-identification and included persons belonging to ethno-cultural transgender identities such as hijras and kinnars as well as gender non binary, gender fluid and gender queer persons from across India. Purposive sampling was done guided by the intersectionality approach and by discussions in the community workshops. For example, the sampling included transgender persons of different classes, castes, regions, cultural identities, gender identities and sexual orientations. An initial set of participants were chosen based on the intersectionality approach from the networks of AS, Sanjana Singh, HR and VP. Subsequently, participants were recruited through snowballing, where each participant was asked to suggest other potential participants and decision to recruit was made by the research team.

The participants were reached *via* email, WhatsApp and by phone call and a pre-interview call was held when needed which helped in rapport building. Prior to initiating data collection, the data collection team was trained in gender sensitivity with focus on interviewing techniques by AS. The IDI and FGD guides were semi-structured and developed in consultation with research team and community members. The data collection was primarily conducted virtually because of restrictions due to the COVID-19 pandemic. Some data were also collected in-person with COVID-19 prevention measures to include the communities that do not have smooth digital access and understanding, such as the ethno-cultural *Hijra/Kinnar* communities.

All the IDIs were audio recorded with consent of participants which was taken over email or verbally and recorded. Transcription was done by SP or consultants who signed a confidentiality agreement and translation was done where necessary to English. All identifiers including but not limited to name, place of residence, name of hospitals or healthcare professionals etc. were omitted from the transcripts to maintain anonymity. The participants were given an option to access their transcripts if they wished to.

### 2.3. Data analysis

A discussion of key observations and ideas after each IDI and FGD was conducted in weekly team meetings. The data analysis uses a two-stage thematic analysis approach (54, 55). Coding was done using Microsoft Excel by SP, HR and CS. In the first stage an open coding approach was used where the codes were generated based on the data. Then the emerged codes were organized into a framework while keeping in mind the theoretical approach of intersectionality. In the second stage all the transcripts were coded in a deductive approach with space to accommodate new inductive codes as well. The code sheets were reviewed and double checked between the coding personnel and a codebook was developed that included codes and subcodes, a definition of the codes and a description of the codes with examples. This was finalized with consensus with all the members of the team. The framework was reviewed by the community in a participatory workshop and with the research team through multiple meetings. Research outputs such as interview audio recording, consent forms, transcribed data, and notes were stored in Sangath's secure cloud storage. Physical documents were stored under lock and key with access to only the investigators.

### 2.4. Use of intersectionality approach

Intersectionality deliberates on how each person's experience is not defined by one identity alone but a host of different identities some giving privileges, while other marginalising depending on the context and the power relations that each identity entail (50, 51). In our study for intersectionality, we looked at the following identities other than the transgender identity: sex assigned at birth, gender identity, sexual orientation, ethno-cultural identity, regional identity, linguistic identity, class, caste and religion. We operationalized the approach through the following steps: (1) intersectional representation in the advisory board and participatory community workshops; (2) recruitment of diverse participants for data collection covering various intersectionalities; (3) ensuring safe space for diverse participants in the data collection; (4) specific questions about intersectionality in the interview guide including the use of an orientation video by AS on intersectionality that was played during the interview; (5) data analysis looking specifically for role of intersectionality.

## 3. Results

22 IDIs and 6 FGDs were conducted in TCC19, and all the transcripts were included in the analysis for this paper. Out of the persons contacted, 8 participants did not take part in the study. The reasons were not getting a response from the potential participants and/or scheduling/interest conflict. Participants included 15 transfeminine, 5 transmasculine and 2 gender non-binary persons. Of the 6 FGDs, 3 were with *Kinnar* communities in three different cities in Central and Northern India. One FGD each were with transfeminine, transmasculine and gender non-binary persons. Data collection was stopped in light of thematic saturation and maximum possible intersectionality that could be accommodated within the project limitations. Thematic saturation and intersectional sample saturation was determined through discussions in team meetings and in a community workshop. A summary of the thematic analysis is provided in Table 1. We focus on findings relevant to mental health in this paper.

**Table 1:** Summary of the thematic analysis.

Theme	Sub Theme
Anxieties due to an inaccessible health system exacerbated	1. Anxieties because of death and suffering 2. Anxiety about hospitalisation 3. Mental distress because of challenges in vaccination 4. Mental healthcare access restricted
COVID related restrictions affected mental health because of unique kinship and social support needs	1. Social isolation during the pandemic 2. Domestic violence 3. Pandemic worsened mental health problems
COVID-19 exacerbated social determinants of mental health	1. Stigma worsened 2. Disrupted livelihoods 3. Difficulty in finding employment
COVID-19 impacted mental health through variable effects on dysphoria and “passability”	1. Dysphoria due to disrupted gender transition 2. Positive effects of change in social interaction

### 3.1. Anxieties due to an inaccessible health system exacerbated

#### 3.1.1. Anxieties because of death and suffering

The COVID-19 pandemic had an adverse impact on the mental health of transgender persons. Many people were anxious about the disease and the uncertainties around it. This was linked to a lack of targeted and systematic communication by the government, especially one that was inclusive of diverse transgender communities. For many participants, seeing death and suffering of family members and other community members exacerbated their mental health problems.

*My father, my mother, my sister, all three of them had COVID. I also saw a lot of my friends suffering a lot. And some of them dying. — A transmasculine person from North India*

#### 3.1.2. Anxiety about hospitalisation

In addition, there was anxiety about getting hospitalized in case of getting infected. This was because of past traumatic experiences in healthcare settings and experiences of others, for example, where trans people where physically examined without consent. A transwoman spoke about feeling anxious about having to get admitted to a hospital. This was also because in India there was mandatory hospitalizations for COVID-19 in the initial phases.

*Next day I got the result; I was positive. The first thing that came to my mind is “okay, as long as I'm at home, it's okay”. But if I have to go to a hospital, and actually get admitted there. I was so anxious of how I am going to be treated as a trans woman. — A transgender woman from Eastern India*

*Some of my friends tried to find affirming medical care. It was terrible, it was very, very difficult and one trans person, for example, was subjected to physical examination without their consent… It had a tremendous effect on my mental health. — A recently graduated trans woman from North India*

Anxiety about hospitalizations was exacerbated with misinformation about the situation during COVID-19. For example, a hijra transwoman shared that she was scared to go to a hospital thinking that she will be killed in the hospital.

*I couldn't go to hospital during COVID because there were a lot of dead bodies, and nobody was going also as they were scared of it… It was widespread belief that even if a healthy person goes to a hospital — they'll kill them also and will file a case also. — A hijra trans woman from North India in an FGD*

Another participant spoke about the mental distress about accessing healthcare because of the negative experiences faced as a transgender person such as neglect.

*So, every time I'm unwell it's not just the physical unwellness that I go through, but also the emotional and mental trauma of being neglected and ignored. — A gender fluid person from North-East India*

#### 3.1.3. Mental distress because of challenges in vaccination

Challenges in accessing vaccines and vaccine information also resulted in mental distress. One participant spoke of how transgender people were “*being shooed away”* from COVID-19 vaccination centers. Many had concerns about the safety of taking the vaccines while being on hormone replacement therapies. Risk of being dead-named in the vaccination certificate and the vaccination center being transphobic were anxiety-provoking. Some vaccination centers were more inclusive.

*I had to get vaccinated under my dead name. Fortunately, there was no gender discrimination — gender binary separation at the vaccination center. There wasn't a separate place for men and women, everyone can sit and get vaccinated at the same place. So, that didn't trigger my dysphoria that much but still I have vaccination certificate [in my dead name] — Trans men in an FGD*

#### 3.1.4. Mental healthcare access restricted

COVID-19 related restrictions also had an impact on mental health by restricting access to mental healthcare. Access to hospitals was reduced because of travel restrictions. Limited mental healthcare was available on a virtual mode but were not found of acceptable quality. In addition, because of challenges with ensuring continued employment because of lockdowns meant that paying for mental health services also became difficult. There were challenges in getting psychiatric medications.

*I couldn't go to the hospital for therapy. And online therapy just didn't work for me. Plus, it was getting a little hard to afford it by myself, as I don't have a job. And so, I just discontinued that. — A non-binary person from South India*

*I have to send the photo of my prescription to some other pharmacist who is going to try and deliver it back home… I am a little anxious about sharing my prescription with too many people. That definitely made it a little difficult to continue — A gender non-binary person from South India*

### 3.2. COVID related restrictions affected mental health because of unique kinship and social support needs

#### 3.2.1. Social isolation during the pandemic

COVID-19 related restrictions impacted mental health of transgender persons adversely. For many people in the trans community support from their chosen family and other kinship structures play an important role in positive mental health. Travel restrictions meant that participants were socially isolated, lonely and felt “*cut off”* from their social networks. Participants were forced to be restricted in their parental homes which often resulted in conflict with family members with regards to their transgender and/or non-binary identity. As educational programmes and employment made a difficult transition during this time to an online mode, having to live with parents added to their mental distress. Being able to meet with others online was very helpful for many people but this was not accessible.

*Queer people, particularly, need to be connected with others in the community. Therefore, the pandemic and the lockdown particularly affected the queer community. Often conversations about gender identity come up when you are stuck at home with the family, for example, even things like “when are you going to get married?” … your freedom to speak to others freely becomes limited. A non-binary person from South India in an FGD*

*The first few months of lockdown when I was at home some of my traumas came back to me…. So being locked, it does at times trigger your traumas which was giving me a lot of nightmares, sleeplessness, stress, anxiety, and a lot of depression. — A transwoman from Eastern India*

#### 3.2.2. Domestic violence

Having to live at the parental home also meant that many people had to come out (disclosing one's trans identity) earlier than planned. Also, many people faced domestic violence.

*Now they may be facing domestic violence and they perhaps even feel trapped in very violent circumstances or sexual assault as well. — A trans woman from South India*

#### 3.2.3. Pandemic worsened mental health problems

Participants spoke of how these multiple factors during the pandemic period “exponentially” worsened their mental health problems. One participant spoke of how their mental health problem “worsened ten times and resulted in two suicide attempts”. Another transwoman spoke of loneliness and worsening of depression.

*There was like a like exponential worsening as they have like went up by at least 10 orders of magnitude. I tried ending my life twice, got admitted in [name of a renowned institution] both the times. — A trans woman from North India*

*My depression has tremendously increased after the pandemic. Bouts of crying, random crying for no specific reason and a terrible amount of loneliness. — A recently graduated trans woman from North India*

### 3.3. COVID-19 exacerbated social determinants of mental health

#### 3.3.1. Stigma worsened

The COVID-19 pandemic exacerbated other social determinants of mental health such as stigma and livelihood. COVID-19 brought new forms of stigma. For example, there was a disinformation campaign in a south Indian city targeting the transgender community, especially the ethnocultural *hijra/ kinnar* community as carriers of COVID with the justification being that they “brought HIV and AIDS”.

*…a lot of people posted posters at railway stations and bus stops saying transgender and Hijra persons brought HIV and AIDS. They are the ones who will bring COVID. — A trans woman from South India*

*… initially when COVID cases were raising, I was witnessing some kind of stigma being around trans people. — A trans woman health professional from South India*

#### 3.3.2. Disrupted livelihoods

Livelihood is an important social determinant of mental health. Employment being moved to an online mode was a difficult transition for many transgender people. Participants shared about working longer hours, even “*15 h a day”*. A transwoman spoke of how working from home increased “pressure” at work which was worsened by a fear of losing her job amidst other layoffs.

*… when you're [working from] home, it's like you have work all the time… the whole company, the clients, everybody pressurizes and everybody's there. The fear is there of losing your job at that time. There were layoffs happening in my office. — A transgender woman from Eastern India*

People from the *Hijra* community who are otherwise dependent on traditional blessing, begging and sex work had major disruptions to their livelihood resulting in financial problems.

*At that time we didn't get anything. It was a troublesome period. At that time, for 2.5–3 months I was in [a western state's city]. Then I travelled to [a city in the central state] on foot as I was facing a lot of financial problems.– A Hijra trans woman from Central North India*

#### 3.3.3. Difficulty in finding employment

Some participants pointed out how the stress was also linked to the uncertainty around employment that many transgender people feel. A transwoman spoke about how challenges in getting a job as a transwoman was worsened by the pandemic and resulted in increased anxiety.

*[The pandemic] has created problems for everyone in terms of employment, especially when you are a trans person and you want employment… [with the pandemic] it has become even more skewed and difficult. So, that has increased my anxieties — around whether I will sustain next year or not [in my job] — A recently graduated trans woman from North India*

### 3.4. COVID-19 impacted mental health through variable effects on dysphoria and “passability”

#### 3.4.1. Dysphoria due to disrupted gender transition

People who got COVID-19 were asked to stop hormone replacement therapy. In addition, difficulty to access gender affirming care (GAC) because of pandemic linked restrictions made people feel dysphoric. Thus, both the COVID-19 infections and the pandemic linked restrictions limited the access to GAC which are vital to the mental health of transgender persons.

*When you are having COVID, you are advised to not take HRT for a month… So, for one month I had to stop that which was a lot of mental issues for me… having dysphoria during the time when you are suffering from COVID was very difficult for me. That added to my anxiety. — A transgender woman from Eastern India*

#### 3.4.2. Positive effects of change in social interaction

One of the aspects that reduces gender dysphoria is the ability to pass as the affirmed gender. Because of this, certain features of the COVID-19 pandemic and its linked restrictions had a positive impact on the mental health of transgender persons. Mask usage, for example, made some people feel more comfortable in social interaction as it felt easier to pass. Similarly, while many people found the shift of jobs to an online mode distressing, others found it more comfortable as cameras capture only the upper half of the body and there is always the freedom to keep cameras switched off. The inability to move out of the house also meant fewer in-person social gatherings which also was found as a positive factor for mental health.

*When the mask is there, it's very easy. It's easier to pass off as a cis woman — A transgender woman from Eastern India*

*I think, I liked it. Because I didn't have to go out much… else it was such a headache to wear that wig and go out. Now I don't have to fully dress up. I just have to be dressed waist upwards. Because everything is on the camera it's very convenient. I don't think people should die of COVID…But I think it'd be nice if COVID remains, and people fear COVID so that this going out is not much. — A trans woman from South India*

## 4. Discussion

This paper examined data from a larger study to examine the mental health impact of the COVID-19 pandemic on transgender persons in India. The study found that the pandemic impacted mental health of transgender persons both negatively and positively through several pathways.

Fear of the pandemic and witnessing the associated suffering had a negative effect on transgender persons (46). Anxiety about potential hospitalizations was an added layer which is linked to a history of stigma and discrimination of transgender persons within healthcare settings (2, 44, 57). This indicates the urgent need to make general healthcare services trans-inclusive and trans-affirmative (58). In addition, there were challenges in accessing mental healthcare during the pandemic. This is in a background of increased mental healthcare need in the community, higher treatment gap and history of trauma within mental healthcare settings (31, 60). These findings point to the need to bring systemic reform in making all healthcare services trans-affirmative including mental healthcare services.

Participants were forced to live in their parental homes with instances of transphobic discrimination and having to come out earlier than expected. Lack of access to in-person peer support, safe spaces and conflict with parents lead to much mental distress and exacerbation of mental health problems (44, 61). Parental support and connectedness play a significant role in the mental health of transgender youth (62, 63). Where parental support is absent, non-parental forms of social support also can reduce risk of mental health problems and self-harm (64). When large scale movement restrictions such as lockdowns are instituted, it is important to develop strategies that allow transgender persons to access the form of social support and spaces that they are most comfortable with.

Challenges with employment and livelihood, such as restrictions to continue *badhaai* and sex work that require physical interaction had a particular impact on mental health of transgender persons (44, 47, 61, 65). This is related to the increased uncertainty and precarity around employment among transgender persons, which predates the pandemic but was further worsened by the pandemic (5, 46, 63, 64).

The study showed how the pandemic brought new forms of stigma to the transgender community, with them being accused of spreading COVID-19. This is in line with literature on trust during pandemics and parallels with South Korea where there was backlash against the LGBTQI + community who were accused of spreading COVID-19 (45, 68, 69). This is in a background of transgender-related stigma which negatively impact mental health for example through higher levels of depression and anxiety (70).

The pandemic impacted mental health of transgender people through gender dysphoria. This was either because of access to GAC being restricted because of pandemic-linked restrictions or because of being advised to do so in the instance of being infected with COVID-19 (44, 46). This points to the need to recognize GAC as an essential health service and the need to ensure its continued availability for better mental health of transgender persons, for example, through telehealth services (68, 69). This is especially important given that GAC is associated with decreased suicide and anxiety in transgender individuals (24, 70). On the other hand, an interesting finding linked to gender dysphoria is the finding of positive impacts of mask usage and virtual meetings. This is because appearance congruence is an important positive factor for mental health (64). This also points to how the transgender mental health, especially gender dysphoria, is intricately linked to how we work as a society.

## 5. Conclusion

The COVID-19 pandemic adversely impacted the mental health of transgender persons in India through several pathways. Firstly, the disease and its associated fear and suffering combined with pre-existing inaccessibility of healthcare and reduced access to mental health care exacerbated mental health problems. Secondly, the unique social support needs of transgender persons were disrupted by the pandemic and its linked restrictions thus affecting mental health. Thirdly, the pandemic impacted mental health because of exacerbations of pre-existing vulnerabilities such as precarious employment and underlying stigma. Finally, gender dysphoria was found to be a key mediating factor in the impact of COVID-19 on mental health with a negative and positive impact.

The study reiterates the prevailing need to make systemic changes to make mental healthcare and general healthcare services trans-inclusive while also recognizing the essential nature of gender affirmative services and the need to continue them even during emergencies and disaster situations (71). In-service training and change of health professional curricula to make it trans-affirmative, changing health regulations such as in insurance policies to make it inclusive of transpersons' needs, especially of people from *hijra/kinnar* community, and revisiting definitions of essential health services are important (60). Government insurance schemes such as Ayushman Bharat in India has already started taking efforts to make them trans-inclusive but more needs to be done and also the same is needed in private health insurance schemes. There is a lot more to be done to provide insurance coverage for mental health services.

In addition, the study brings some crucial insights in the intersection of gender and mental health. On one hand, the study brings out how public health emergencies can exacerbate vulnerabilities — through precarious livelihood, unsupported kinship structures and new forms of underlying stigma. On the other it also shows how the lived mental health experience of transgender persons is intricately linked to the way work, travel and housing is structured in our society, which determine experiences of gender dysphoria and therefore points to the structural nature of the linkage between mental health and gender.

Thus, this paper sheds light on the systemic and structural determinants of mental health, how health emergencies exacerbate them and uniquely impact marginalized communities. Being cognizant of these will help program managers and policy makers across the world make structurally informed interventions to improve mental health vulnerability.

As this manuscript is a product of analysis of data from another study there were several limitations such as limited data. Future studies are warranted that can explore further several questions unearthed by this study. This includes questions such as: health access and its impact on mental health, kinship and social support structures of transgender persons and its role in mental health, precarious employment and its relationship with mental health and gender dysphoria in online spaces. Further, there is a need for a systematic effort to adapt the minority stress model to the transgender community in the Indian context.

## Data Availability

The datasets presented in this article are not readily available because they were collected from a marginalised community. Requests to access the datasets should be directed to the corresponding author.
